# Signal mining and risk analysis of Alprazolam adverse events based on the FAERS database

**DOI:** 10.1038/s41598-024-57909-y

**Published:** 2024-03-29

**Authors:** Feng Huang, Xiao San, Qingqian Liu, Haohao Zhu, Wenrong Xu

**Affiliations:** 1 Department of Clinical Laboratory, Kunshan Maternity and Children’s Health Care Hospital, Kunshan, 215300 Jiangsu China; 2 Department of Clinical Laboratory, The First People’s Hospital of Kunshan Affiliated With Jiangsu University, Kunshan, 215300 Jiangsu China; 3 Mental Health Center of Jiangnan University, Wuxi Central Rehabilitation Hospital, Wuxi, 214151 Jiangsu China; 4 Jiangsu Key Laboratory of Medical Science and Laboratory Medicine, School of Medicine, Jiangsu University, Zhenjiang, 212013 Jiangsu China

**Keywords:** Alprazolam, FAERS, Real-world data analysis, Adverse events, Adverse drug reaction, Psychiatric disorders, Risk factors

## Abstract

This study aims to evaluate the safety of Alprazolam by analyzing the FAERS database, provide data analysis for monitoring adverse drug reactions. This research encompasses adverse event (AE) reports related to Alprazolam from the first quarter of 2004 to the second quarter of 2023. Four signal mining and analysis methods were utilized, including Reporting Odds Ratio (ROR), Proportional Reporting Ratio (PRR), Bayesian Confidence Propagation Neural Network (BCPNN), and Empirical Bayesian Geometric Mean (EBGM). Further exploration was conducted regarding patient characteristics and types of AEs. A total of 23,575 AE reports in which Alprazolam was the primary suspect drug were collected, identifying 347 Preferred Term (PT) signals and 27 System Organ Classes (SOCs). The number of AE reports increased annually, especially in 2015, 2018, 2019, and 2020. The main affected groups were females and the age range of 18 to 45. Psychiatric disorders, Nervous system disorders, and Gastrointestinal disorders were the most common the organ system in which the AEs occurred. There is a certain risk of drug abuse and suicide with Alprazolam. Most notably, several AEs not recorded in the Alprazolam leaflet appeared among the top 30 PTs in signal strength, including but not limited to Benzodiazepine drug level abnormal, Acquired amegakaryocytic thrombocytopenia, Cutaneous T-cell dyscrasia, and Coronary No-reflow Phenomenon. For the first time, AEs related to the cardiovascular system and platelet function were unveiled. The severe AE reports that resulted in "hospitalization" and "death" accounted for 30.96% and 21.86%. This study highlights the risks of suicide and misuse of Alprazolam. Other potential severe or fatal AEs, such as those related to the cardiovascular system, platelet function, and others, require further research to determine their precise mechanisms and risk factors.

## Introduction

Evaluating and regulating drug safety is pivotal to preserving public health. As pharmaceuticals are broadly consumed, it's crucial to report and monitor adverse events (AEs) to timely identify potential risks and safety concerns. A cornerstone of the drug monitoring ecosystem is the Adverse Event Reporting System (FAERS) of the U.S. Food and Drug Administration (FDA)—a nationwide database archiving AE information related to drug consumption^1,2^.

Alprazolam, as a commonly prescribed intermediate-acting benzodiazepine (BZD), primarily amplifies the inhibitory neurotransmitter Gamma-Aminobutyric Acid (GABA) system, thereby depressing the excitability of the central nervous system^3^. Recognized for its pronounced anti-anxiety effects, rapid onset, and efficacy against insomnia, Alprazolam has seen extensive use in medical practice. From 2015 to 2018, there were 71,481 dispensings of Alprazolam to 6772 people in Australia. Following a policy intervention in 2017, the overall dispensing of Alprazolam decreased by 51.2%, but the prescribing approvals increased by 17.5%^4^. Moreover, studies have indicated that short-term BZD usage can alleviate anxiety and insomnia symptoms in depression patients during the initial phase of antidepressant treatment, not only hastening relief from severe depression but also potentially enhancing the sustained efficacy of antidepressants^5,6^.

However, the broad clinical application of Alprazolam doesn't come without its slew of AEs and latent risks. At clinical doses, beyond its widely acknowledged sedative, hypnotic, anxiolytic, and muscle relaxant effects, Alprazolam may induce psychomotor disorders and cognitive degeneration. It's also possibly correlated with increased risks of Alzheimer's disease, strokes, and malignant brain tumors^7,8^. Additionally, due to its unique pharmacokinetics (high potency, short half-life, rapid absorption and withdrawal effects), Alprazolam manifests a higher propensity for abuse, potentially triggering withdrawal syndromes more severe than other BZDs, thereby negatively impacting health and quality of life^9^.

To better understand the safety profile and inherent risks of Alprazolam, and to deliver a more comprehensive insight to underpin prudent medicinal decisions, this study delves into AE data associated with Alprazolam in FAERS. It aims to unearth safety signals and risk factors through a meticulous analysis, thoroughly probing patterns, trends, and correlated factors of Alprazolam's AEs.

## Materials and methods

### Data source

The data for this study was sourced from the FAERS database. FAERS collects spontaneous safety reports and post-marketing clinical study reports related to drug use both within and outside the United States. This study selected data from the first quarter of 2004 to the second quarter of 2023. Analysis was performed using MySQL, and after deduplication, AE reports where Alprazolam was the primary suspected drug were obtained.

### Data processing

Using "Alprazolam" as the keyword for retrieval from the database, we obtained details including personal information, drug information, AEs, and primary diseases. Following the FDA-recommended method for removing duplicate reports, we select the PRIMARYID (Primary Identification), CASEID (Case Identification), and FDA_DT fields from the DEMO table. We sort by CASEID, FDA_DT, and then PRIMARYID. For reports with the same CASEID, we retain the one with the largest FDA_DT value. FDA_DT refers to the FDA received date of the adverse event report. The rationale is that the report with the most recent received date likely contains the most up-to-date and complete information for that case. Secondly, for reports where both CASEID and FDA_DT are the same, we retain the one with the largest PRIMARYID value. PRIMARYID is a unique identifier assigned to each report. By keeping the report with the largest PRIMARYID, we aim to preserve the most complete data. Since the first quarter of 2019, each quarterly data package has included a list of deleted reports. After data deduplication, we remove reports based on the CASEID listed in the deleted reports list. The "Medical Dictionary for Regulatory Activities" (MedDRA) version 23.0 was used for AE terminologies. This included preferred SOC (System Organ Class) and PT (Preferred Term) for classifying and expressing AEs^10^.

### Data analysis

Signal detection for AEs is essentially determining whether the reporting frequency of a specific AE for the target drug is higher than expected, thus establishing a statistical association between the drug and that specific AE. This study employed the Reporting Odds Ratio (ROR)^11^, Proportional Reporting Ratio (PRR) ^12^, Bayesian Confidence Propagation Neural Network (BCPNN)^13,14^, and the Empirical Bayesian Geometric Mean (EBGM)^15^ through SAS 9.4 software. The ROR helps mitigate biases in events with fewer reports. The PRR stands out for its greater specificity compared to ROR. BCPNN is adept at combining and cross-validating multi-source data. The MGPS is particularly effective in identifying signals from infrequent events. This study utilizes a blend of ROR, PRR, BCPNN, and MGPS to capitalize on their individual strengths, enhancing the scope of detection and validation from diverse angles. This integrated approach aids in more accurately identifying safety signals, reducing false positives through cross-validation and refining detection of rare adverse reactions by adjusting thresholds and variance. These methods are based on a 2 × 2 contingency table, as shown in Table 1. The formulas for each method and the conditions that satisfy signal generation are presented in Table 2.

**Table 1:** Contingency table.

	Target AEs	Non-target AEs	Total
Alprazolam	a	b	a + b
Non-Alprazolam	c	d	c + d
Total	a + c	b + d	N = a + b + c + d

**Table 2:** ROR, PRR, BCPNN, and EBGM methods, formulas, and thresholds.

Method	Formula	Threshold
ROR	${\text{ROR}}=\frac{(a/c)}{(b/d)}=\frac{ad}{bc}$ ${\text{SE}}({\text{lnROR}})=\sqrt{\left(\frac{1}{{\text{a}}}+\frac{1}{{\text{b}}}+\frac{1}{{\text{c}}}+\frac{1}{{\text{d}}}\right)}$ $95\mathrm{\%CI}={{\text{e}}}^{{\text{ln}}({\text{ROR}})\pm 1.96\sqrt{ \left(\frac{1}{{\text{a}}}+\frac{1}{{\text{b}}}+\frac{1}{{\text{c}}}+\frac{1}{{\text{d}}}\right)}}$	a ≥ 3 and 95% CI (lower limit) > 1
PRR	${\text{PRR}}=\frac{a/(a+b)}{c/(c+d)}$ SE (lnPRR) = $\sqrt{\frac{1}{a}-\frac{1}{a+b}+\frac{1}{c}-\frac{1}{{\text{c}}+{\text{d}}}}$ $95\mathrm{\%CI}$=${e}^{{\text{ln}}({\text{PRR}})\pm 1.96\sqrt{ \frac{1}{a}-\frac{1}{a+b}+\frac{1}{c}-\frac{1}{{\text{c}}+{\text{d}}}}}$	a ≥ 3 and 95% CI (lower limit) > 1
BCPNN	IC = ${log}_{2}\frac{p(x,y)}{p(x)p(y)}={log}_{2}\frac{a(a+b+c+d)}{(a+b)(a+c)}$ E (IC) = ${log}_{2}\frac{(a+\gamma 11)(a+b+c+d+\alpha )(a+b+c+d+\beta )}{(a+b+c+d+\gamma )(a+b+\alpha 1)(a+c+\beta 1)}$ V (IC) = $\frac{1}{{(ln2)}^{2}}\left\{\left[\frac{\left(a+b+c+d\right)-a+\gamma -\gamma 11}{\left(a+\gamma 11\right)\left(1+a+b+c+d+\gamma \right)}\right]+\left[\frac{\left(a+b+c+d\right)-\left(a+b\right)+\alpha -\alpha 1}{\left(a+b+\alpha 1\right)\left(1+a+b+c+d+\alpha \right)}\right]+\left[\frac{\left(a+b+c+d\right)-\left(a+c\right)+\beta -\beta 1}{\left(a+c+\beta 1\right)\left(1+a+b+c+d+\beta \right)}\right]\right\}$ $\gamma =\gamma 11\frac{(a+b+c+d+\alpha )(a+b+c+d+\beta )}{(a+b+\alpha 1)(a+c+\beta 1)}$ *IC-2SD* = *E(IC)-2* $\sqrt{V(IC)}$	IC025 > 0
EBGM	${\text{EBGM}}=\frac{a(a+b+c+d)}{(a+c)(a+b)}$ $95\mathrm{\%CI}={{\text{e}}}^{{\text{ln}}({\text{EBGM}})\pm 1.96\sqrt{ \left(\frac{1}{{\text{a}}}+\frac{1}{{\text{b}}}+\frac{1}{{\text{c}}}+\frac{1}{{\text{d}}}\right)}}$	EBGM05 > 2

## Results

### Constituent ratio of yearly data

In this study, from January 1, 2004 to June 30, 2023, a total of 19,932,732 AE reports were obtained. Among them, 23,575 reports suspected Alprazolam as the primary drug. After analysis using the ROR, PRR, BCPNN, and EBGM methods, 347 signals on the PT level and 27 signals on the SOC level were detected. Female subjects dominated the AE reports for Alprazolam, accounting for 56.53%, while males accounted for 35.84%. Reports covered patients of all age groups, with the 18 to 45 age bracket having the highest proportion at 27.86%. Reports of AEs related to Alprazolam have shown an increasing trend year by year from 2004 to 2023. Notably, in 2015, 2018, 2019, and 2020, there was a significant increase in the number of reports, accounting for 8.97%, 10.52%, 13.86%, and 13.76% of the total reports, respectively. Consumers were the primary reporters, making up 35.37% of the total reports. The majority of reports came from the USA (54.43%), followed by France (18.57%) and Italy (9.96%). The outcomes of AEs showed that the most common were Hospitalization—Initial or Prolonged (30.96%) and Death (21.86%), suggesting that Alprazolam might be associated with some serious AEs (Table 3).

**Table 3:** Constituent ratio of yearly data of AEs related to Alprazolam.

Factors	Number of events (%)
Gender	
Female	13,326 (56.53)
Male	8450 (35.84)
Unknown	1799 (7.63)
Age	
< 18	704 (2.99)
18–45	6569 (27.86)
45–65	5937 (25.18)
65–75	1911 (8.11)
≥ 75	1853 (7.86)
Unknown	6601 (28.00)
Reporter	
Consumer	8339 (35.37)
Pharmacist	4658 (19.76)
Physician	7061 (29.95)
Other health professionals	2971 (12.60)
Unknown	470 (1.99)
Lawyer	76 (0.32)
Reported countries	
United States	12,833 (54.43)
France	4377 (18.57)
Italy	2347 (9.96)
Brazil	752 (3.19)
Japan	436 (1.85)
Report year	
2004	175 (0.74)
2005	212 (0.90)
2006	195 (0.83)
2007	287 (1.22)
2008	278 (1.18)
2009	271 (1.15)
2010	630 (2.67)
2011	434 (1.84)
2012	535 (2.27)
2013	551 (2.34)
2014	931 (3.95)
2015	2114 (8.97)
2016	1764 (7.48)
2017	1639 (6.95)
2018	2480 (10.52)
2019	3268 (13.86)
2020	3245 (13.76)
2021	1789 (7.59)
2022	1795 (7.61)
2023	982 (4.17)
Serious outcomes	
Death	5153 (21.86)
Disability	451 (1.91)
Hospitalization—initial or prolonged	7298 (30.96)
Life-Threatening	1158 (4.91)
Adverse event occurrence time—medication date (days)	
0–30	4158 (17.64)
31–60	92 (0.39)
61–90	77 (0.33)
91–120	46 (0.20)
121–150	34 (0.14)
151–180	35 (0.15)
181–360	106 (0.45)
> 360	482 (2.04)

### Risk signal analysis results

In Table 4, the SOCs with many signals included: Psychiatric disorders, General disorders and administration site conditions, Nervous system disorders, Injury, poisoning and procedural complications, Gastrointestinal disorders. Among them, General disorders and administration site conditions, Injury, poisoning and procedural complications, Respiratory, thoracic and mediastinal disorders, Musculoskeletal and connective tissue disorders, Vascular disorders had a large number of reports.

**Table 4:** The signal strength of AEs of Alprazolam at the SOC level.

System organ class	SOC code	Case reports	ROR (95% CI)	PRR (95% CI)	χ^2^	IC (IC025)	EBGM (EBGM05)
Psychiatric disorders	10,037,175	19,010	5.44 (5.35–5.53)	4.34 (4.29–4.40)	51,536.65	2.11 (2.09)	4.32 (4.25)
General disorders and administration site conditions	10,018,065	11,490	0.83 (0.81–0.85)	0.86 (0.84–0.87)	337.35	−0.22 (− 0.25)	0.86 (0.84)
Nervous system disorders	10,029,205	10,865	1.74 (1.70–1.77)	1.63 (1.60–1.66)	2903.67	0.71 (0.68)	1.63 (1.60)
Injury, poisoning and procedural complications	10,022,117	10,139	1.38 (1.35–1.41)	1.33 (1.30–1.35)	911.54	0.41 (0.38)	1.33 (1.30)
Gastrointestinal disorders	10,017,947	3303	0.48 (0.46–0.49)	0.50 (0.48–0.52)	1804.74	− 1.00 (− 1.05)	0.50 (0.48)
Respiratory, thoracic and mediastinal disorders	10,038,738	2898	0.79 (0.76–0.82)	0.80 (0.77–0.82)	158.98	− 0.33 (− 0.38)	0.80 (0.77)
Cardiac disorders	10,007,541	2830	1.36 (1.31–1.42)	1.35 (1.30–1.40)	265.26	0.43 (0.38)	1.35 (1.30)
Investigations	10,022,891	2698	0.55 (0.53–0.57)	0.56 (0.54–0.58)	978.76	− 0.83 (− 0.89)	0.56 (0.54)
Musculoskeletal and connective tissue disorders	10,028,395	1636	0.39 (0.37–0.41)	0.41 (0.39–0.43)	1499.56	− 1.30 (− 1.37)	0.41 (0.39)
Skin and subcutaneous tissue disorders	10,040,785	1448	0.34 (0.32–0.35)	0.35 (0.33–0.37)	1861.21	− 1.52 (− 1.59)	0.35 (0.33)
Vascular disorders	10,047,065	1243	0.74 (0.70–0.78)	0.74 (0.70–0.78)	115.23	− 0.43 (− 0.51)	0.74 (0.70)
Eye disorders	10,015,919	1201	0.79 (0.74–0.83)	0.79 (0.75–0.83)	69.26	− 0.34 (− 0.43)	0.79 (0.75)
Product issues	10,077,536	1139	0.95 (0.90–1.01)	0.95 (0.90–1.01)	2.48	− 0.07 (− 0.15)	0.95 (0.90)
Metabolism and nutrition disorders	10,027,433	1095	0.64 (0.61–0.68)	0.65 (0.61–0.69)	213.34	− 0.62 (− 0.71)	0.65 (0.61)
Infections and infestations	10,021,881	1018	0.24 (0.23–0.26)	0.25 (0.24–0.27)	2345.19	− 1.97 (− 2.06)	0.25 (0.24)
Social circumstances	10,041,244	865	2.44 (2.28–2.61)	2.43 (2.27–2.59)	726.22	1.27 (1.17)	2.42 (2.26)
Immune system disorders	10,021,428	794	0.94 (0.88–1.01)	0.94 (0.88–1.01)	2.66	− 0.08 (− 0.19)	0.94 (0.88)
Renal and urinary disorders	10,038,359	673	0.44 (0.41–0.48)	0.45 (0.41–0.48)	470.14	− 1.16 (− 1.27)	0.45 (0.41)
Hepatobiliary disorders	10,019,805	518	0.73 (0.67–0.80)	0.73 (0.67–0.80)	51.17	− 0.45 (− 0.58)	0.73 (0.67)
Ear and labyrinth disorders	10,013,993	479	1.43 (1.30–1.56)	1.42 (1.30–1.56)	60.34	0.51 (0.37)	1.42 (1.30)
Blood and lymphatic system disorders	10,005,329	398	0.30 (0.28–0.34)	0.31 (0.28–0.34)	631.01	− 1.70 (− 1.84)	0.31 (0.28)
Neoplasms benign, malignant and unspecified (incl cysts and polyps)	10,029,104	379	0.18 (0.16–0.19)	0.18 (0.16–0.20)	1455.74	− 2.47 (− 2.62)	0.18 (0.16)
Surgical and medical procedures	10,042,613	259	0.26 (0.23–0.29)	0.26 (0.23–0.29)	558.37	− 1.95 (− 2.13)	0.26 (0.23)
Congenital, familial and genetic disorders	10,010,331	230	0.96 (0.84–1.09)	0.96 (0.84–1.09)	0.43	− 0.06 (− 0.25)	0.96 (0.84)
Pregnancy, puerperium and perinatal conditions	10,036,585	224	0.65 (0.57–0.75)	0.66 (0.58–0.75)	40.68	− 0.61 (− 0.80)	0.66 (0.58)
Reproductive system and breast disorders	10,038,604	164	0.23 (0.20–0.27)	0.23 (0.20–0.27)	427.43	− 2.11 (− 2.34)	0.23 (0.20)
Endocrine disorders	10,014,698	141	0.74 (0.62–0.87)	0.74 (0.62–0.87)	13.30	− 0.44 (− 0.68)	0.74 (0.62)

In Table 5, among the top 30 PTs by report count, most were common adverse reactions for psychiatric drugs. Drug abuse, Drug dependence, Overdose, and Withdrawal syndrome had high occurrence rates. The suicide risk has also been detected, including Completed suicide, Suicide attempt, and Suicidal ideation.

**Table 5:** The top 30 adverse events of Alprazolam ranked by case reports.

SOC	PTs	Case reports	ROR (95% CI)	PRR (95% CI)	χ^2^	IC (IC025)	EBGM (EBGM05)
Psychiatric disorders	Drug abuse	3836	39.45 (38.15–40.78)	37.53 (36.36–38.75)	129,025.80	5.14 (5.09)	35.51 (34.34)
Injury, poisoning and procedural complications	Toxicity to various agents	1943	8.74 (8.35–9.14)	8.54 (8.17–8.93)	12,807.38	3.07 (3.01)	8.44 (8.07)
Psychiatric disorders	Completed suicide	1342	12.23 (11.58–12.91)	12.03 (11.40–12.69)	13,340.48	3.55 (3.47)	11.83 (11.20)
Nervous system disorders	Somnolence	1340	5.34 (5.06–5.64)	5.27 (4.99–5.55)	4608.54	2.38 (2.30)	5.23 (4.96)
Psychiatric disorders	Drug dependence	1133	4.87 (4.59–5.17)	4.81 (4.54–5.10)	3409.00	2.25 (2.17)	4.79 (4.51)
Injury, poisoning and procedural complications	Overdose	1089	3.93 (3.70–4.17)	3.89 (3.66–4.12)	2327.56	1.95 (1.86)	3.87 (3.64)
Psychiatric disorders	Anxiety	1071	2.90 (2.73–3.08)	2.87 (2.71–3.05)	1309.60	1.52 (1.43)	2.87 (2.70)
Psychiatric disorders	Sopor	1065	64.03 (60.10–68.22)	63.16 (59.33–67.24)	59,311.78	5.77 (5.68)	57.57 (54.04)
Psychiatric disorders	Suicide attempt	1031	13.75 (12.92–14.63)	13.58 (12.77–14.43)	11,772.48	3.72 (3.63)	13.31 (12.51)
Injury, poisoning and procedural complications	Intentional overdose	955	12.11 (11.35–12.91)	11.97 (11.23–12.76)	9434.09	3.54 (3.45)	11.77 (11.03)
Nervous system disorders	Coma	922	15.47 (14.48–16.52)	15.29 (14.33–16.32)	12,039.16	3.88 (3.78)	14.96 (14.01)
Psychiatric disorders	Insomnia	824	2.40 (2.24–2.57)	2.38 (2.23–2.55)	661.74	1.25 (1.15)	2.38 (2.22)
Injury, poisoning and procedural complications	Intentional product misuse	769	5.38 (5.01–5.78)	5.34 (4.97–5.73)	2691.51	2.40 (2.29)	5.30 (4.93)
General disorders and administration site conditions	Feeling abnormal	750	2.34 (2.18–2.52)	2.33 (2.17–2.50)	569.39	1.21 (1.11)	2.32 (2.16)
General disorders and administration site conditions	Withdrawal syndrome	625	12.82 (11.84–13.88)	12.73 (11.76–13.77)	6625.72	3.62 (3.50)	12.50 (11.54)
Psychiatric disorders	Confusional state	601	2.90 (2.68–3.14)	2.88 (2.66–3.12)	738.67	1.52 (1.40)	2.88 (2.65)
Cardiac disorders	Cardiac arrest	587	5.43 (5.01–5.90)	5.40 (4.98–5.86)	2090.41	2.41 (2.29)	5.36 (4.94)
Cardiac disorders	Cardio-respiratory arrest	530	9.43 (8.66–10.28)	9.38 (8.61–10.21)	3910.91	3.19 (3.06)	9.25 (8.49)
Nervous system disorders	Loss of consciousness	523	3.17 (2.91–3.46)	3.16 (2.90–3.44)	769.72	1.65 (1.52)	3.15 (2.89)
Respiratory, thoracic and mediastinal disorders	Respiratory arrest	513	13.45 (12.32–14.68)	13.37 (12.25–14.58)	5751.50	3.68 (3.55)	13.11 (12.01)
Nervous system disorders	Tremor	491	2.27 (2.07–2.48)	2.26 (2.07–2.47)	343.81	1.17 (1.04)	2.25 (2.06)
General disorders and administration site conditions	Drug interaction	487	2.41 (2.20–2.64)	2.40 (2.20–2.62)	397.87	1.26 (1.13)	2.40 (2.19)
Psychiatric disorders	Intentional self-injury	479	15.73 (14.36–17.23)	15.64 (14.29–17.12)	6409.29	3.89 (3.76)	15.29 (13.96)
Injury, poisoning and procedural complications	Poisoning deliberate	463	54.86 (49.88–60.33)	54.53 (49.61–59.95)	22,422.49	5.51 (5.37)	50.33 (45.76)
General disorders and administration site conditions	Drug withdrawal syndrome	425	3.26 (2.97–3.59)	3.25 (2.96–3.57)	660.02	1.69 (1.55)	3.24 (2.94)
Psychiatric disorders	Suicidal ideation	380	3.24 (2.93–3.58)	3.23 (2.92–3.57)	581.80	1.68 (1.53)	3.22 (2.91)
Psychiatric disorders	Panic attack	379	8.20 (7.41–9.08)	8.16 (7.38–9.03)	2354.32	2.99 (2.84)	8.07 (7.29)
Nervous system disorders	Depressed level of consciousness	378	7.48 (6.76–8.28)	7.45 (6.73–8.24)	2087.76	2.86 (2.71)	7.38 (6.66)
Psychiatric disorders	Agitation	377	3.84 (3.47–4.25)	3.83 (3.46–4.23)	783.27	1.92 (1.77)	3.81 (3.44)
Cardiac disorders	Tachycardia	269	2.39 (2.12–2.69)	2.38 (2.11–2.69)	215.53	1.24 (1.07)	2.38 (2.11)

In Table 6, the top 30 PTs by signal strength included many adverse reactions not recorded in the Alprazolam leaflet. Among them, Benzodiazepine drug level abnormal, Acquired amegakaryocytic thrombocytopenia, Postnatal growth restriction, Prescription form tampering, Papillary muscle disorder ranked the top five, being new potential adverse reactions. Additionally, even though Cutaneous T-cell dyscrasia, Sarcomatoid carcinoma, Pseudophaeochromocytoma, and Coronary no-reflow phenomenon had fewer reports, their signal strengths were strong, necessitating further attention.

**Table 6:** The top signal strength of AEs of Alprazolam ranked by EBGM at the PTs level.

SOC	PTs	Case reports	ROR (95% CI)	PRR (95% CI)	χ^2^	IC (IC025)	EBGM (EBGM05)
Investigations	Benzodiazepine drug level abnormal	7	447.58 (170.36–1175.89)	447.54 (170.36–1175.72)	1834.59	2.96 (1.73)	263.67 (100.36)
Blood and lymphatic system disorders	Acquired amegakaryocytic thrombocytopenia	6	182.68 (73.73–452.64)	182.67 (73.73–452.58)	843.15	2.75 (1.53)	142.30 (57.43)
Pregnancy, puerperium and perinatal conditions	Postnatal growth restriction	4	182.68 (60.13–555.00)	182.67 (60.13–554.95)	562.10	2.28 (0.83)	142.30 (46.84)
Social circumstances	Prescription form tampering	122	161.08 (132.06–196.47)	160.82 (131.88–196.12)	15,482.91	5.98 (5.69)	128.70 (105.51)
Cardiac disorders	Papillary muscle disorder	4	159.84 (53.44–478.14)	159.84 (53.44–478.09)	505.095	2.28 (0.84)	128.07 (42.81)
Social circumstances	Victim of chemical submission	46	140.80 (102.31–193.76)	140.72 (102.27–193.62)	5230.19	5.07 (4.61)	115.51 (83.94)
Investigations	Postmortem blood drug level abnormal	19	135.01 (82.30–221.45)	134.97 (82.29–221.38)	2086.20	4.09 (3.39)	111.62 (68.05)
Blood and lymphatic system disorders	Cutaneous T-cell dyscrasia	4	116.25 (40.06–337.36)	116.24 (40.06–337.32)	386.70	2.26 (0.86)	98.51 (33.95)
Neoplasms benign, malignant and unspecified (incl cysts and polyps)	Sarcomatoid carcinoma	6	109.61 (46.10–260.60)	109.60 (46.10–260.57)	551.18	2.72 (1.54)	93.71 (39.41)
Nervous system disorders	Peripheral nerve paresis	6	103.69 (43.76–245.67)	103.68 (43.76–245.64)	524.99	2.71 (1.54)	89.35 (37.71)
Psychiatric disorders	Withdrawal catatonia	15	95.92 (55.74–165.05)	95.90 (55.74–165.00)	1224.94	3.76 (2.99)	83.52 (48.54)
Immune system disorders	Anamnestic reaction	6	93.57 (39.72–220.40)	93.56 (39.72–220.37)	479.30	2.70 (1.54)	81.75 (34.70)
Endocrine disorders	Pseudophaeochromocytoma	7	91.34 (41.37–201.67)	91.33 (41.37–201.64)	547.24	2.88 (1.79)	80.04 (36.25)
Cardiac disorders	Coronary no-reflow phenomenon	3	87.19 (26.09–291.30)	87.18 (26.09–291.28)	224.91	1.94 (0.39)	76.84 (23.00)
Psychiatric disorders	Paramnesia	8	85.25 (40.77–178.29)	85.25 (40.77–178.26)	587.70	3.02 (2.00)	75.33 (36.02)
Social circumstances	Chemical submission	15	84.88 (49.53–145.47)	84.87 (49.53–145.43)	1097.52	3.74 (2.97)	75.04 (43.79)
Social circumstances	Substance abuser	9	68.51 (34.45–136.24)	68.50 (34.45–136.22)	540.71	3.12 (2.16)	61.97 (31.16)
Psychiatric disorders	Sopor	1065	64.03 (60.10–68.22)	63.16 (59.33–67.24)	59,311.78	5.77 (5.68)	57.57 (54.04)
Nervous system disorders	Adrenergic syndrome	5	58.13 (23.27–145.21)	58.12 (23.27–145.19)	257.31	2.45 (1.22)	53.36 (21.36)
Nervous system disorders	Glossopharyngeal nerve disorder	3	58.12 (17.83–189.53)	58.12 (17.83–189.51)	154.38	1.92 (0.40)	53.36 (16.37)
Injury, poisoning and procedural complications	Poisoning deliberate	463	54.86 (49.88–60.33)	54.53 (49.61–59.95)	22,422.49	5.51 (5.37)	50.33 (45.76)
Surgical and medical procedures	Drug withdrawal maintenance therapy	4	48.25 (17.46–133.33)	48.25 (17.46–133.32)	172.10	2.20 (0.85)	44.94 (16.26)
Investigations	Coma scale	4	44.87 (16.28–123.66)	44.87 (16.28–123.65)	160.30	2.19 (0.85)	41.99 (15.24)
Respiratory, thoracic and mediastinal disorders	Pneumonitis aspiration	12	44.10 (24.57–79.16)	44.09 (24.57–79.14)	472.78	3.33 (2.50)	41.31 (23.01)
Investigations	Benzodiazepine drug level increased	5	43.79 (17.70–108.37)	43.79 (17.70–108.36)	195.67	2.42 (1.20)	41.05 (16.59)
Psychiatric disorders	Drug use disorder	256	43.56 (38.37–49.44)	43.41 (38.26–49.26)	9934.24	5.14 (4.95)	40.72 (35.87)
Social circumstances	Immobilisation prolonged	6	41.70 (18.26–95.24)	41.70 (18.26–95.22)	223.73	2.60 (1.47)	39.20 (17.17)
Psychiatric disorders	Mixed anxiety and depressive disorder	11	40.66 (22.10–74.79)	40.65 (22.10–74.77)	400.01	3.22 (2.36)	38.28 (20.81)
Respiratory, thoracic and mediastinal disorders	Bradypnoea	108	39.15 (32.24–47.55)	39.10 (32.20–47.48)	3778.54	4.79 (4.51)	36.90 (30.38)
Injury, poisoning and procedural complications	Oesophagitis chemical	3	38.36 (11.97–123.00)	38.36 (11.97–122.99)	102.98	1.88 (0.39)	36.25 (11.30)

## Discussion

Benzodiazepines (BZDs), derived from 1,4-benzodiazepine, play a pivotal role in the alleviation and treatment of emotional anxiety, hyperactive reactions, insomnia, and epilepsy. They are currently the most widely used and longest-standing drugs for insomnia treatment. The primary mechanism of these drugs is by acting on the reticular structure of the brainstem and the limbic system, enhancing the affinity between the inhibitory neurotransmitter GABA and its respective receptors, thus inducing a suppressive effect on the central nervous system. Alprazolam, introduced to the U.S. market in 1981, is a medium-acting benzodiazepine. It possesses various effects such as anti-anxiety, anti-convulsion, and anti-depression, making it one of the most extensively used BZDs. Past researches, via case reports, systematic reviews, prescription monitoring, and clinical trials, have primarily studied and evaluated the adverse reactions related to Alprazolam.

Previous case reports on Alprazolam's adverse reactions mainly include allergic reactions, addiction, withdrawal responses, short-term memory loss, sleepwalking, muscle weakness, mental disorders, frequent urination, bloody lactation, gum overgrowth, agitation, abnormal behaviors, lethal overdose, dose-dependent orgasmic disorders, and acute angle-closure glaucoma^16^. Additionally, studies indicated that women taking Alprazolam during the first three months of pregnancy may increase the risk of congenital anomalies^17–19^. Nursing mothers consuming Alprazolam might lead to withdrawal symptoms and mild drowsiness in infants. Consuming Alprazolam during pregnancy might be linked to inguinal hernia, fetal deformities, and neonatal withdrawal syndrome, while its use during breastfeeding could cause mild drowsiness and withdrawal symptoms in infants. This research deeply analyzes the AE report data of Alprazolam to gain a more comprehensive understanding of its safety and the overview of AEs, providing a foundation for further risk management and clinical practice.

### Report trends and patient characteristics analysis

This study delved into the AE reports from 2004 to 2023, especially focusing on reports where Alprazolam was the primary suspected drug. The results unveiled a series of noteworthy phenomena and trends. Firstly, in terms of the number of AE reports, there's an annual increase in reports related to Alprazolam. Specifically, there were marked surges in 2015, 2018, 2019, and 2020. This trend hints at the rising societal and medical institutional concerns about the safety of Alprazolam. It might also suggest a broadening usage scope of this drug or its association with more complex and severe adverse reactions.

Secondly, the gender and age distribution in the reports is intriguing. Females dominate the AE reports for Alprazolam^20^. This could imply that females might be more susceptible to Alprazolam's adverse effects or are more inclined to report these events. The most common age group in these reports is between 18 to 45 years, typically considered the most socially and professionally active cohort. Hence, these adverse reactions could severely impact their work and social lives.

### Existing adverse reactions

The AEs primarily associated with Alprazolam center around Psychiatric disorders, encompassing Drug abuse, Completed suicide, and Drug dependence. Issues of Drug abuse and Drug dependence hint at the potential addictiveness of Alprazolam, posing risks to patient safety. Of particular concern are suicide-related events such as Completed suicide and Suicidal ideation. This aligns with previous research, emphasizing the need for heightened vigilance regarding a patient's mental well-being while on Alprazolam, coupled with corresponding preventive measures.

Beyond Psychiatric disorders, Nervous system disorders also stake a claim in Alprazolam's list of adverse reactions. This includes Somnolence, Coma, and Confusional state. Such reactions could be life-threatening, especially when driving or operating machinery. Injury, poisoning, and procedural complications emerge as another area of concern, especially for long-term Alprazolam users. Such irregularities could lead to grave health issues, necessitating routine monitoring.

### New adverse reactions and potential mechanisms

Risk signal analyses unveil various aspects warranting further research. While there's a plethora of signals regarding General disorders and administration site conditions, and Injury, poisoning and procedural complications, the medicine's documentation doesn't explicitly cite these adverse reactions. This suggests that both doctors and patients might lack adequate awareness and vigilance towards these latent risks.

Signal strength analyses further expose several potential adverse reactions not documented in Alprazolam's instructions. These newfound adverse reactions pertain to the cardiovascular system and platelet function, including Benzodiazepine drug level abnormal, Acquired amegakaryocytic thrombocytopenia, and Papillary muscle disorder. The mechanisms underlying these reactions remain elusive, but they might involve drug metabolism, interactions, and intricate physiological processes. For instance, Benzodiazepine drug level abnormal might stem from interactions between Alprazolam and other drugs, affecting its metabolism—a facet demanding further illumination^21^. Meanwhile, Acquired amegakaryocytic thrombocytopenia might be linked to an undiscovered correlation between Alprazolam and platelet functionality, necessitating in-depth experimentation and study^22^.

This research also identified rare but significant adverse reactions, namely Cutaneous T-cell dyscrasia, Sarcomatoid carcinoma, Pseudophaeochromocytoma, and the Coronary no-reflow phenomenon. For instance, Cutaneous T-cell dyscrasia might arise from Alprazolam's impact on the immune system^23^, and Sarcomatoid carcinoma could suggest that the drug indirectly affects biological pathways associated with cancer growth^24^. Pseudophaeochromocytoma might simulate some symptoms of pheochromocytoma by impacting the sympathetic nervous system. The Coronary No-reflow Phenomenon, a blood flow obstruction involving coronary arteries, may relate to Alprazolam's indirect effects on the cardiovascular system, particularly with high dosages or long-term use^25,26^.

Even though these adverse reactions are relatively less reported, given their severity and potential life-threatening nature, they warrant further research and attention. These findings signal the need for vigilance, regarding not only common side effects of Alprazolam but also these rare yet potentially severe reactions.

### Limitations

There were several limitations regarding to the FAERS database^27^. The primary limitation of this study is that all data come from a voluntary AE reporting database. The spontaneous reporting system suffers from a significant underreporting issue, therefore the results do not reflect the full picture of the actual adverse reactions occurring. The study did not consider medication dosage data, making it impossible to interpret the results in the context of drug dosage, thus presenting certain limitations. Hence, these results should be viewed as a preliminary understanding of Alprazolam's safety concerns and should not replace more systematic and rigorous clinical studies. Besides, without specific data to directly correlate the rise in adverse event reports with increased usage, it's difficult to confirm this relationship definitively. Nonetheless, these initial findings undeniably set a direction and foundation for deeper future research and discussions.

## Conclusion

In summary, this research provides vital safety information for the clinical use of Alprazolam. It unveils various concerns and risks that demand further scrutiny. The risks of suicide and abuse remain areas of significant concern. The cardiovascular system, platelet function, and other serious and potentially fatal issues require further studies to determine their precise mechanisms and risk factors. Given Alprazolam's widespread use in treating anxiety, insomnia, and other symptoms, it's imperative to deeply understand and address these issues. Future studies should examine Alprazolam's safety concerns more comprehensively and meticulously, aiming for more precise clinical guidance.

## Data Availability

The dataset generated during and analyzed during the current study are available from the corresponding author on reasonable request.
